# How does reimbursement status affect smoking cessation interventions? A real-life experience from the Eastern Black Sea region of Turkey

**DOI:** 10.18332/tid/100412

**Published:** 2019-01-22

**Authors:** Dilek Karadoğan, Özgür Önal, Yalçın Kanbay

**Affiliations:** 1 Department of Chest Diseases, School of Medicine, Recep Tayyip Erdoğan University, Rize, Turkey; 2 Department of Public Health, School of Medicine, Süleyman Demirel University, Isparta, Turkey; 3 Department of Psychiatric Nursing, School of Health Science, Çoruh University, Artvin, Turkey

**Keywords:** reimbursement, smoking cessation, insurance coverage, treatment adherence, quit success

## Abstract

**INTRODUCTION:**

In the last decade, outpatient smoking cessation clinics (SCCs) in Turkey have been extended countrywide. Initially, only counseling was covered under health insurance. In 2011 and 2015, free varenicline and bupropion preparations were distributed to SCCs, periodically. In the current study we aimed to compare outcomes between the free and paid medication periods.

**METHODS:**

Patients applied to the local SCC in a secondary health care unit between June 2014 and June 2017. They were evaluated for SC interventions and had phone visits after their third month; these records were included in the study. Patients were grouped and evaluated according to medication’s reimbursement status: free medication period (FP) and paid medication period (PMP).

**RESULTS:**

In total, 733 patients applied to the SCC, 77.7% of them had applied during the FP. Analyses were made involving 417 patients who had records of third-month phone visit. Mean age of the patients was 44.0±13.7 years with the majority of patients (65%) being male. Sociodemographic characteristics of patients in both groups were not statistically different, while the percentage of patients with comorbid diseases was lower in the FP group (p<0.05). Treatment choices were different— the bupropion-prescribed group’s rate was similar in both periods (53.5% in PMP vs 52.0% in FP), however varenicline was mostly prescribed in the FP (35.8% vs 14.1%) while nicotine replacement therapy was mostly prescribed in the PMP (32.4% vs 12.1%) (p<0.05). Patients who used the advised treatment for at least 30 days (treatment adherent) and the rate of quitters at the third month were higher in FP (p<0.05) from univariate analysis, however these differences were not statistically significant when a multivariate analysis was performed.

**CONCLUSIONS:**

Our study showed that the free medication period increased the quit attempts but the increased in treatment adherence and quit success of the participating smokers was not obvious.

## INTRODUCTION

Smoking is one of the major causes of preventable death in the world. In Turkey, its prevalence is 27.1% among adults over 15 years of age, with 41.5% for males and 13.1% for females^1^. According to the recent Global Adult Tobacco Survey, 46% of Turkish smokers tried to quit smoking at least once in the last year, of whom 73.4% had tried without assistance^2^. Tobacco use was classified as a disorder associated with substance use for the first time in 1994^2^, and this disorder needs to be treated accordingly^3^— as quit success increases from 7% to 15.7% with assistance^4^. The most effective method for cessation is a combination of cognitive behavioral therapy and pharmacotherapy^5^. There are three types of first choice medical treatment in Turkey: nicotine replacement therapy (NRT), bupropion, and varenicline^6^. After Turkey signed the World Health Organization Framework Convention for Tobacco Control (WHO FCTC) in 2004, tobacco control has become of fundamental importance in the country. The number of smoking cessation clinics has been increasing annually; until 2002 there were only 25 SCCs in the country while in 2018 there were 417^7^. SCCs provide motivation and appropriate cessation methods for smokers and play an important role in cessation success. In 2011, for the first time 300000 bupropion and varenicline preparations were distributed to SCCs by the Turkish Ministry of Health (MoH). They repeated this initiative for a second time in 2015 and continued for 1.5 years. During other periods, smokers who wanted to quit smoking would pay for prescribed cessation medication themselves^8^. There was one study in Turkey that evaluated the outcomes of these two implementations between years 2011 and 2012 and found lower quit success at 6 months among the free-drug user group compared to the group of patients who had to pay for their treatment^9^. There is, however, no study that examined the outcome of the Turkish MoH second free medication distribution period that started in 2015.

Previous studies have proven the effectiveness of smoking cessation free-of-charge interventions^10-12^ . However, the number of studies that compare the effects of free and paid interventions in real-life settings are limited, both in Turkey and worldwide^9,13^. In this study we aimed to evaluate the free smoking cessation distribution period in 2015 compared with other periods, retrospectively. To our knowledge, our study is the second to compare these two periods in Turkey, giving useful data to show the gaps that must be filled to improve tobacco control policies.

## METHODS

### Setting and samples

The study population was chosen from patients over 18 years of age who applied to a secondary level government hospital’s smoking cessation clinic, located in the Eastern Black Sea region of Turkey, between 14 July 2014 and 30 June 2017. At that time, this SCC was staffed by one pulmonologist (certified for SC counselling by the Turkish MoH), one nurse, and one medical secretary. It accepted patients one day a week for SC counselling. Patients were able to make their appointments by calling a national appointment’s line via the Turkish MoH. At their first visit, patients gave information on their medical history, had physical examinations, laboratory tests (complete blood cell count and renal and liver function tests), chest x-ray, pulmonary function tests and an electrocardiography. In their second visit, all their information was entered into the Turkish MoH’s tobacco addiction treatment monitoring system— an online system started in April 2015. Before that date, patient data were archived in files manually. Any doctor who has a certificate to work in SCCs can access this online system via a unique username and password. The system requires six steps when entering patient information. The first step asks for sociodemographic data (name, ID number, phone number, date of birth, gender, education level, occupation). The second step asks questions for the Fagerström test for nicotine dependence. The third step includes questions about their smoking history (date of first cigarette/length of smoking habit, number of previous quit attempts, date of last quit attempt, and any methods used for previous quit attempts). The fourth step evaluates comorbid conditions, pregnancy/lactation status for females, and disease/medication history. The fifth step evaluates symptoms such as: cough, sputum production, reduction of exercise capacity, dyspnea, anxiety, chest pain, and blood pressure. If possible, exhaled carbon monoxide level is included, as well as reason for consultation, if needed. The last step gives the total Fagerström test score automatically and the planned treatment choice is recorded (pharmacotherapy and/ or behavioral). Planned cessation date is recorded and an appointment date is given for the second visit. All the data are then saved in the system. In their second visit, often seven or fourteen days later, quit status and adverse reactions are evaluated, and appointments planned for other visits. Any new data are recorded on the online system.

Patients were manually grouped by their filed signed informed consent for proactive counselling and treatment choice in monthly groups. Their names, ID numbers, phone numbers, and prescribed medications were also recorded manually in a separate notebook. At 3 months after their initial visit, they were called by phone and asked questions in three areas: number of days medication was used, adverse reactions, and quit status.

Inclusion criteria were being older than 18 years of age, having been evaluated as a clinically and psychiatrically appropriate candidate for smoking cessation, the pharmacotherapy started, and completing phone calls during the third month. Those who did not fall into this category, or could not be reached by phone, were excluded.

### Grouping of coverage period

**Figure 1:**
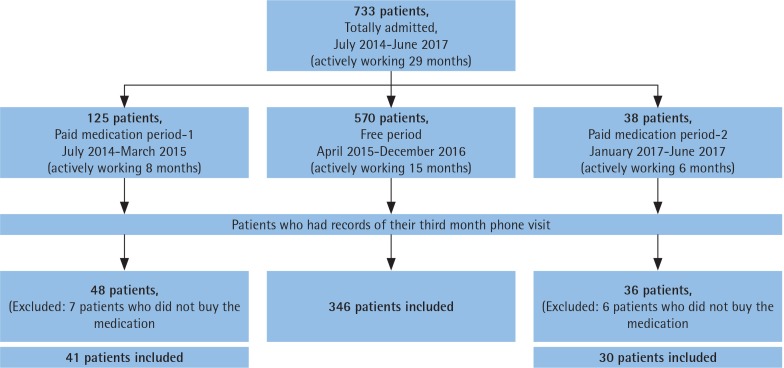
Admission number of patients according to the periods and the patient recruitment

Figure 1 shows all three periods of the study: the first period was July 2014–April 2015, second period was May 2015–December 2016, and the third period was January 2017–June 2017. In the second period only, smoking cessation medications or SCMs (varenicline and bupropion) were distributed to SCCs free-ofcharge.

### Confounding variables

Patients were started on bupropion, varenicline, or combined nicotine replacement therapy (cNRT)—nicotine patch and nicotine gum. Smokers that used the prescribed medication at least 30 days were grouped as ‘treatment adherent’; the others who used less than 30 days were grouped as ‘treatment non-adherent’. Patients who declared that they had quit after the planned quit date in their third month phone call were classified as ‘quitters’; those who were continuing to smoke or relapsed were ‘non-quitters’.

### Statistical analysis

First, descriptive statistics were used for all three periods. Admission rates to the SCC were expressed as graphs. For univariate and multivariate analysis those periods were examined under two groups: free medication period (FP) and paid medication period (PMP). Afterwards, for the continuous variables a t-test was used, while a chi-squared test was used for categorial variables. Backward logistic regression (LR) was used to determine the effect of confounding variables on both periods and also effect of variables on treatment adherence and quit success.

Ethical approval was obtained from the local ethics committee, and permission for the study was also obtained from the General Secretary of the hospital.

## RESULTS

### Admission rates and treatment choices according to periods

**Figure 2:**
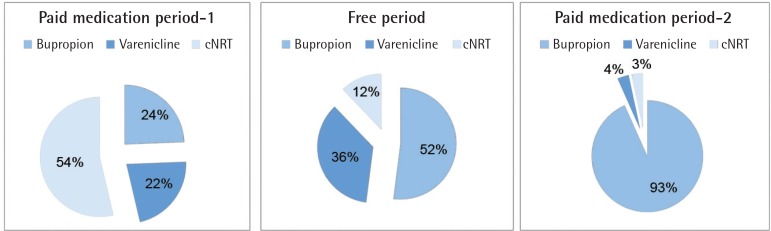
Distribution of started treatment choices according to the periods

In total, 733 patients had applied to the SCC during the 29-month period (Figure 1). Among them, 77.7% had applied during the free medication distribution period (FP). Furthermore, the mean monthly admission number was highest in the free period (38 patients, while it was 15 for the first free counselling only period and 6 for the last). Different distributions of treatment choices in all periods are shown in Figure 2. In the free period, mostly bupropion/varenicline were advised, while in the first period cNRT was prescribed, and for the last period bupropion was the most frequently prescribed treatment choice.

### Patient characteristics according to the reimbursement periods

For the study, 417 patients met the inclusion criteria and therefore statistical analysis was done on their data. There were 346 patients in FP and 71 patients in PMP (Table 1). Mean age of the study population was 44.0±13.7 years, and in terms of sociodemographic characteristics both periods were similar. However, the rate of patients with comorbid disease was higher in PMP, as was the number of NRT users. The rate of bupropion users was similar. Patients who sought treatment during the free period had higher Fagerström test scores, a higher rate of starting varenicline, higher rates of treatment adherence, and higher mean control visits. The rate of successful quitters was also higher in FP (p<0.05) (Table 1).

**Table 1:** Characteristics of the study population

*Variables*	*Total ( 417 )*	*Paid medication period ( 71 )*	*Free period ( 346 )*	*p*
**Age, mean±SD**	44.0±13.7	42.7±12.5	44.3±13.9	0.343
**Gender Male, n (%)**	271 (65.0)	50 (70.4)	221 (63.9)	0.35
**Education level, n (%)**				0.72
Less than 5-years	157 (37.6)	27 (38.0)	130 (37.6)	
5-years primary schooling	44 (10.6)	10 (14.0)	34 (9.8)	
3-years secondary schooling	136 (32.6)	22 (30.9)	114 (32.9)	
3-years high schooling	80 (19.2)	12 (16.9)	68 (19.7)	
**Occupation, n (%)**				0.17
Blue collar worker or farmer	120 (28.7)	22 (31.0)	98 (28.3)	
				
White collar worker	102 (24.4)	18 (25.4)	84 (24.3)	
Housewife or not working	100 (23.9)	14 (19.7)	86 (24.9)	
**Presence of comorbid disease, n (%)**	129 (30.9)	29 (40.8)	100 (28.9)	**0.047**
**Fagerström score, mean±SD**	6.47±2.02	5.74±2.16	6.62±1.96	**0.002**
**≥6,** n (%)	293 (70.2)	41 (66.1)	252 (72.8)	0.28
**Started medical treatment, n (%)**				**<0.0001**
Varenicline	134 (32.1)	10 (14.1)	124 (35.8)	
				
Bupropion	218 (52.2)	38 (53.5)	180 (52.0)	
cNRT	65 (15.5)	23 (32.4)	42 (12.1)	
**Treatment use duration, mean±SD**	20.1±18.3	16.3±17.1	20.8±18.5	0.058
**Treatment adherent (≥30 days), n (%)**	162 (38.8)	20 (28.2)	142 (41)	**0.043**
**Control visit number, mean±SD**	1.40±1.10	0.74±0.93	1.54±1.09	**<0.0001**
**Presence of adverse reactions, n (%)**	103 (24.7)	15 (21.1)	88 (25.4)	0.44
**Quit smoking, n (%)**	149 (35.7)	18 (25.4)	131 (37.9)	**0.045**

A multivariate analysis with Backward LR test was performed among all variables in Table 1; older age, higher control visit number, absence of comorbid disease, varenicline and bupropion use compared to cNRT were associated factors with FP (Table 2).

**Table 2:** Statistically significantly associated characteristics with free period*

	*Multivariate analysis (Backward LR)*		
	*OR*	*95% CI*	*p*
Age (per 1 age increment)	1.032	1.006–1.058	0.014
Control visit number (per 1 visit increment)	3.136	2.032–4.838	<0.001
Absence vs Presence of comorbidity	2.37	1.254–4.490	0.008
cNRT	1		
Varenicline	5.948	2.437–14.517	0.023
Bupropion	2.291	1.112–4.680	<0.001

Method: Backward Stepwise (likelihood ratio): –2 Log likehood: 302.889; Cox and Snell R^2^: 0.170; Negelkerke R^2^: 0.284, Omnibus test of model coefficients: p<0.001. *Adjusted with all of the variables in Table 1: age, gender, education level, job, presence of comorbid diseases, Fagerström score, started medication, treatment adherence, control visit number, adverse reaction status and quit status.

### Factors affecting treatment adherence

Varenicline and bupropion use were associated with increased treatment adherence compared to cNRT; varenicline users’ treatment adherence was also higher than bupropion users. An absence of adverse reactions and a higher number of control visits were also associated with higher treatment adherence, both in univariate and multivariate analysis. However, the FP group’s treatment adherence was significantly higher only in univariate analysis (Table 3).

**Table 3:** The affector factors on treatment adherence in univariate and multivariate analysis

	*Univariate analysis*			*Multivariate analysis (Backward LR)*		
	*OR*	*95% CI*	*p*	*OR*	*95% CI*	*p*
**Started treatment choice**						
cNRT	1			1		
Bupropion	2.184	1.120–4.261	**0.022**	3.038	1.460–6.322	**0.003**
Varenicline	4.645	2.316–9.319	**0.001**	5.444	2.526–11.684	**0.001**
*Bupropion*	1			1		
*Varenicline*	2.127	1.371–3.298	**0.001**	2.558	1.384–4.725	**0.003**
**Adverse reaction (absence compared to presence)**	1.765	1.092–2.854	**0.020**	3.015	1.735–5.238	**0.001**
**Control visit number (per 1 visit increase)**	1.718	1.399–2.109	**0.001**	1.694	1.356–2.117	**0.001**
**Free period compared to not-free period**	1.775	1.014–3.107	**0.045**	–	–	**NS**

Method: Backward Stepwise (likelihood ratio): –2 Log likelihood: 475.755; Nagelkerke R^2^: 0.154; Omnibus test of model coefficients: p=0.000. Analyses were adjusted for age, gender, education level, job, comorbid disease, Fagerström test, treatment choice, control visit, adverse reactions, quit status and reimbursement periods.

### Factors affecting quit success

Older age (middle age and older) were associated with higher quit success in both analyses. Varenicline use was also associated with higher quit success in multivariate analysis. More control visits were associated with higher quit success in univariate analysis only. Treatment adherence was associated with increased quit success as well. Being in the FP group was associated with increased quit success only in univariate analysis (Table 4).

**Table 4:** The affector factors on quit success in univariate and multivariate analysis

	*Univariate analysis*			*Multivariate analysis (Backward LR)*		
	*OR*	*95% CI*	*p*	*OR*	*95% CI*	*p*
**Age groups** (years)						
15–44	1			**1**		
45–64	2.495	1.210–5.418	**0.013**	**1.901**	**1.080–3.346**	**0.026**
≥65	2.083	0.998–4.347	0.051	**3.791**	**1.574–9.131**	**0.003**
**Started treatment choice**						
Bupropion	1			1		
Varenicline	1.291	0.723–2.305	0.387	1.984	1.071–3.674	**0.029**
cNRT	0.791	0.430–1.454	0.450	1.613	0.780–3.335	0.197
**Control visit number** (per 1 visit increase)	1.461	1.210–1.765	**0.001**	–	–	**NS**
**Treatment adherent compared to non– adherent**	3.449	2.266–5.247	**0.001**	**3.056**	**1.918–4.867**	**0.001**
**Free period compared to not–free period**	1.794	1.007–3.195	**0.047**	–	–	**NS**

Method: Backward Stepwise (likelihood ratio): –2 Log likelihood: 458.124; Nagelkerke R^2^: 0.177; Omnibus test of model coefficients: p=0.000. Analyses were adjusted for age, gender, education level, job, comorbid disease, Fagerström test, treatment choice, control visit, adverse reactions, treatment adherence, quit status and reimbursement periods.

Drug distribution differed between periods, especially for varenicline and cNRT, and only bupropion had similar rates of use in both periods; therefore, we evaluated bupropion users’ quit success across both periods. Patients in the FP using bupropion had higher quit success compared to those using it during the NFP (OR=3.554; 95% CI: 1.322–9.557; p<0.05). Mean bupropion use in the FP was also higher compared to PMP; 19.7±15.6 and 11.2±14.9, respectively (p<0.05).

## DISCUSSION

This study showed that a period of free SC medication disbursement increased the number of attempts to quit by patients visiting the smoking cessation clinic, but the effect on treatment adherence and on quit success was not statistically significantly different. It also provided evidence that the reimbursement status of the medication had an effect on the clinician’s treatment choice. The overall quit rate at the third month was found to be 35%, and the treatment adherence rate was 38%. Treatment adherence was found to be the biggest factor in quit success. An absence of side effects, increased control visits, varenicline use versus bupropion, and being in the free medication period group all increased treatment adherence.

It has been suggested that the financial cost of SC treatments can act as a barrier to those seeking support; the effectiveness of free interventions was previously proven in clinical trials^14^. However, the need for studies tracking real-life experiences without additional quitting incentives for participants has been noted^13^. Therefore, our study was designed to give a real-life experience on the topic.

The efficacy of free-of-charge smoking cessation interventions has been proven in studies worldwide^11,12^. Additionally, in a recent study, proof of the cost-effectiveness and need for covering the cost smoking cessation interventions under insurance has been reported^10^. In another study, full financial interventions directed at smokers were found to increase the proportion of smokers who attempted to quit, used smoking cessation treatments, and succeeded in quitting^14^. However, these studies mostly took place in developed countries. There was a study that evaluated the nationwide smoking cessation intervention periods’ outcomes separately^15^. Also, in another study that was the first to compare the outcomes of the free and paid medication periods of Turkey between years 2011 and 2012, it was found that smokers who used free drugs had lower quit rates at 6 months compared to those who paid for the medication, 14.8% versus 27.3%^9^. Our study is second in Turkey to directly compare the short-term outcomes of the free medication period with the period in which only counseling is included at no cost. However, both the previous study^9^ and our study have some limitations that decrease their power. In both studies, different rates of confounder factors such as frequency of type of medication, time of usage, mean age, mean Fagerström scores of both periods, limit the ability to directly evaluate the free period’s effect on the outcome. We, however, performed multivariate logistic regression analysis with Backward LR test to evaluate the factors associated with free period, which was not done in the other study.

In our results, one of the important points is that admission rate was highest during the FP. It is known that each year most smokers intend to quit, but only 5% of them succeed because 73.4% of smokers who intend to quit try it on their own, without support^1^. Through free medical interventions, smokers who want to quit can have a higher chance of success. A free period encourages those smokers who are making a genuine effort to quit. In an international study, during the free reimbursement period telephone counselling was 10 times more common than during paid periods. Similarly, that study covered two paid periods and one free period. In the paid period following the year when medication was reimbursed, though the percentage of smokers in the population did drop by about 3%, the number of patients who signed up for SC programs was also at the lowest level of the entire study^16^. In our study, the mean number of monthly patient admissions to the SCC was 15 in the first PMP; this decreased to 6 in the second PMP following the FP. Admissions were highest during the free period with 38 patients monthly. The decrease in enrollment following the free period may be due to smokers anticipating another reimbursement period and thus deciding to wait in case it occurs.

Another interesting finding was that only 38% of patients were treatment adherent; 41% in FP and 28.2% in PMP (p<0.05). In multivariate analysis the difference between periods was not statistically significant. In both periods, medication usage durations were still a problem. There is a need to evaluate the reasons behind inadequate treatment usage and premature discontinuation. In other studies, reported explanations included smoking relapses, experiencing medication-related side effects, and believing that the medication was no longer needed^17^. These could also be issues within our study population, however we did not record that data specifically in this study. More professional, full-time SC interventions and close follow-ups are required to educate and give more intensive support and information to patients—not only about adverse reactions to treatment but about how the mechanism works to affect SC. However, in univariate analysis the FP was also found to increase the treatment adherence, so there is a chance that cost does play a role in early treatment cessation. Medication cost was found by Iranian researchers^18^ to have an effect on varenicline users. Also, in the same study they found higher quit rates in the group that used varenicline over 6 weeks.

In our study, only the bupropion user groups’ number was available for comparisons and multivariate analysis according to reimbursement status. Patients in the FP using bupropion had both higher quit success and treatment usage duration compared to those using it during the NFP. Also, treatment adherence of cNRT users was lowest because of cost.

In terms of clinician response to the FP, the data showed that prescribed treatment choices were different in each period. In the first PMP, for example, mostly cNRT was prescribed. In the FP, bupropion and varenicline had the higher rates of prescription, while cNRT had the lowest rate. Finally, in the last NFP, bupropion had the highest rate. One of the reasons for this result might be the changes in the clinician’s experience. At first, due to the potential risks of drug-related side effects, mostly cNRTs were preferred, because they are thought to cause fewer complications. Another explanation may be the cost of the medications. Bupropion and varenicline have monthly packages, and their monthly costs were between 72–110 Turkish lira (TL) for bupropion and 195–262 TL for varenicline, during the time of the study. For that reason, nicotine patches and gums were offered, because that form of treatment had weekly packaging, which allowed patients to at least try it to see whether it will work. Weekly nicotine patch prices were 36–48 TL and nicotine gum prices were 20–24 TL; therefore, the monthly cost of cNRT was approximately 200 TL, higher than for bupropion. In the last PMP, bupropion was mostly offered—that is strongly related to the cost of the drugs, because the monthly cost of bupropion was lowest. Physicians also observed that offering cNRT caused premature discontinuation; because of the cost, patients only bought one pack of patches and gum (adequate for only 1 week). Therefore, in the last period bupropion was chosen. In the FP, mostly free medications of varenicline and bupropion were started, for obvious reasons. Thus, our results also show that the cost of the medication is a potential barrier in cessation interventions. Excluding this barrier will positively affect SC interventions, as discussed previously^19^.

Benli et al.^20^ conducted a study in 2011, during the first free medication period, and found the quit rate to be 25.9% at the third month. Longer medication usage was associated with higher quit rates. In the same study, the quit rate at the 6th month was found to be down to 15.8%, and they suggested that one of the reasons for the low success was the free medication distribution itself. Smokers without the intent and willingness to quit had just rushed to get medication when it was available. This theory is backed up by other research—in evaluations after one of the free distribution periods (which can last up to 1.5 years), it was found that some of the patients had not even started to use the medication prescribed. In another recent national study, Önur et al.^21^ found the quit rate during a paid medication period to be 25% at the 6th month; and that each member of the quitter group had followed up with control visits at least once. It is worth mentioning that the mean participant ages were different between these studies: 35 years for the Benli et al. study and 45 years for the Önür et al. This variable may be one of the factors for the difference in quit rates; as previous research has shown that older patients are more likely to have symptoms from long-term smoking and are thus more motivated to quit^22^. In our study as well, older age groups had higher quit rates in multivariate analysis, as did varenicline users and those who continued their medication for at least 30 days. The difference in quit rates between the various medications is likely due to the metabolism and mechanism of the drugs, as reported previously^23^. In univariate analysis, higher numbers of control visits and being in the FP, as opposed to the PMP, also resulted in statistically significant higher quit rates.

### Limitations

This study is the second to compare the free and paid periods of medication disbursement within the recent Turkish tobacco control policy. It is a study of real-life experiences from the same clinic and pulmonologist, but as it was performed at a specific location it may not be generalizable globally. Furthermore, evaluations of quit status depended on self-reports and is one of the limitations, however, previous studies have shown that discrepancies between self-reported and biochemically verified smoking status are minimal among the general population, even in special patients groups^24-26^. Also, heterogeneous distribution of patient characteristics such as age, comorbid condition status and started treatment choice may have an influence on the results. Additionally, due to the retrospective design of the study all of the patients could not be included because of incomplete data.

## CONCLUSIONS

The current study makes it apparent that free SC medication distribution, in the Eastern Black Sea region of Turkey, increased quit attempts but the increase in treatment adherence and quit success of the participating smokers was not obvious. One of the reasons for this result could be the heterogeneous characteristic distribution of patients between the groups. Only the patients who started bupropion were available for multivariate analysis, therefore only those patients were evaluated according to their reimbursement status and among them significantly higher treatment adherence and quit success rates were detected in FP, compared to patients who used bupropion in PMP. More studies with homogeneous patient distribution for minimizing confounding factors are required to compare the outcomes of both periods. Interestingly, it was also discovered that the cost of the medications directly affected the clinicians’ treatment choices because SCMs can be costly for some smokers. There is a need at all times to cover SCMs under insurance, like other treatment choices, so that smokers can apply when they feel ready to quit. Additionally, SCC teams should prioritize closer follow-ups and spend more time on pretreatment education and motivation. In this way clinicians can maximize the positive effects of the free period and increase quit success rates consistently.

## CONFLICTS OF INTEREST

Authors have completed and submitted the ICMJE Form for Disclosure of Potential Conflicts of Interest and none was reported.
