# Corrigendum: How does reimbursement status affect smoking cessation interventions? A real-life experience from the Eastern Black Sea region of Turkey

**DOI:** 10.18332/tid/112274

**Published:** 2019-09-13

**Authors:** Dilek Karadoğan, Özgür Önal, Yalçın Kanbay

**Affiliations:** 1 Department of Chest Diseases, School of Medicine, Recep Tayyip Erdoğan University, Rize, Turkey; 2 Department of Public Health, School of Medicine, Süleyman Demirel University, Isparta, Turkey; 3 Department of Psychiatric Nursing, School of Health Science, Çoruh University, Artvin, Turkey

**Keywords:** reimbursement, smoking cessation, insurance coverage, treatment adherence, quit success

**Corrigendum on:**

How does reimbursement status affect smoking cessation interventions? A real-life experience from the Eastern Black Sea region of Turkey By Dilek Karadoğan, Özgür Önal, Yalçın Kanbay Tobacco Induced Diseases, Volume 17, Issue January, Pages 1-9, Publish date: 22 January 2019, DOI: https://doi.org/10.18332/tid/100412

There was an omission of the first paragraph in the **Methods** section that was supported by one extra reference, hence the numbers in the reference list and the superscripted numbers, n, in the text, following reference 14, should now read n+1.

The paragraph is the following:

## Design of the study

This study was planned to evaluate the effect of reimbursement status on smoking cessation interventions, retrospectively, by using a study population, that applied at the free smoking-cessation medication distribution period, comprising groups that obtained the smoking cessation medications out of their own pocket. The free smoking-cessation medications distributed group’s treatment adherence and quit success status were previously studied and published^14^. For this current study that group was used as a control group for evaluating a new research question. Ethical approval was obtained from the local ethics committee (number:78646441-050.01.04-date:19.04.2018), and permission for the study was also obtained from the General Secretary of the hospital.

The additional reference is:

14. Karadoğan D, Önal O, Say Şahin D, Kanbay Y, Alp S, Şahin U. Treatment adherence and short-term outcomes of smoking cessation outpatient clinic patients. Tob Induc Dis. 2018;16(August). doi:10.18332/tid/94212

